# Large-scale gene gains and losses molded the NLR defense arsenal during the *Cucurbita* evolution

**DOI:** 10.1007/s00425-021-03717-x

**Published:** 2021-09-24

**Authors:** Giuseppe Andolfo, Cristina S. Sánchez, Joaquìn Cañizares, Maria B. Pico, Maria R. Ercolano

**Affiliations:** 1 Department of Agricultural Sciences, University of Naples “Federico II”, Portici, NA Italy; 2 Instituto de Conservación y Mejora de la Agrodiversidad Valenciana, Universitat Politècnica de València, Valencia, Spain

**Keywords:** Diversifying selection, Orthology relations, Phylogeny, R-genes, Transcriptomes

## Abstract

**Main conclusion:**

Genome*-*wide annotation reveals that the gene birth–death process of the *Cucurbita* R family is associated with a species-specific diversification of TNL and CNL protein classes.

**Abstract:**

The Cucurbitaceae family includes nearly 1000 plant species known universally as cucurbits. *Cucurbita* genus includes many economically important worldwide crops vulnerable to more than 200 pathogens. Therefore, the identification of pathogen-recognition genes is of utmost importance for this genus. The major class of plant-resistance (R) genes encodes nucleotide-binding site and leucine-rich repeat (NLR) proteins, and is divided into three sub-classes namely, TIR-NB-LRR (TNL), CC-NB-LRR (CNL) and RPW8-NB-LRR (RNL). Although the characterization of the NLR gene family has been carried out in important *Cucurbita* species, this information is still linked to the availability of sequenced genomes. In this study, we analyzed 40 de novo transcriptomes and 5 genome assemblies, which were explored to investigate the *Cucurbita* expressed-NLR (eNLR) and NLR repertoires using an ad hoc gene annotation approach. Over 1850 NLR-encoding genes were identified, finely characterized and compared to 96 well-characterized plant R-genes. The maximum likelihood analyses revealed an unusual diversification of CNL/TNL genes and a strong RNL conservation. Indeed, several gene gain and loss events have shaped the *Cucurbita* NLR family. Finally, to provide a first validation step *Cucurbita*, eNLRs were explored by real-time PCR analysis. The NLR repertories of the 12 *Cucurbita* species presented in this paper will be useful to discover novel R-genes.

**Supplementary Information:**

The online version contains supplementary material available at 10.1007/s00425-021-03717-x.

## Introduction

*Cucurbita* is an important genus of the Cucurbitaceae family that encloses independently domesticated species native to Central and South America (Castellanos-Morales et al. 2018). The taxonomy of this genus is still much debated, as the number of accepted species varies from 13 to 30 (https://plants.usda.gov/ and https://www.itis.gov/). A burst of morphological transformations along the Cucurbitaceae evolutionary history was found after early genome duplication events (Guo et al. 2020). *Cucurbita* species can be divided into two main groups: (i) mesophytes, annual or short-lived perennial herbaceous vines; and (ii) xerophytes, perennials growing in arid zones (Kates et al. 2017; Castellanos-Morales et al. 2018). Cultivated *Cucurbita* species belong to the first group, of which five domesticated crops (*C. argyrosperma*, *C. ficifolia*, *C. maxima*, *C. moschata*, and *C. pepo*) are grown worldwide for their edible fruits and seeds (Castellanos-Morales et al. 2018), variously known as squash, pumpkin or gourd. The earliest known evidence of the domestication of *Cucurbita* is dated 10,000 calendar years B.P. (Renner and Schaefer 2016; Kates et al. 2017; Smith 1997), preceding the domestication of other crops (e.g., for maize is started 8,700 calendar years B.P.). At least six independent domestication events from distinct wild ancestors were identified in domesticated *Cucurbita* (Sanjur et al. 2002). Certainly, domestication and further breeding contributed to narrowing the genetic base of the species, making the *Cucurbita* crops susceptible to existing pests and diseases (Román et al. 2020). This vulnerability, along with the increasing interest in developing new, environmentally friendly *Cucurbita*-resistant cultivars, steers the identification and the study of structural and functional mechanisms of the evolution of pathogen-recognition genes. The wild cucurbits represent a valuable genetic resource for crop improvement (Khoury et al. 2020), in which novel disease-resistance genes could be identified and transferred into modern *Cucurbita* cultivars (Capuozzo et al. 2017). To date, the information on the defense arsenal of wild *Cucurbita* species is very limited, therefore, its exploration is an important goal for developing cultivars.

Basically, the plants have developed two pathogen-recognition layers to repel their attacks (PTI: PAMP-triggered immunity and ETI: Effector-triggered immunity) (Andolfo and Ercolano 2015). The pathogen perception mainly occurs through the recognition of effectors via plant disease-resistance (R) proteins. The major class of R-genes is the nucleotide oligomerization domain (NOD)-like receptors (NLRs), which encode nucleotide-binding site (NB) and leucine-rich repeat (LRR) gene families (Michelmore et al. 2013). NLRs are generally divided into three sub-classes, based on different N-terminus architecture, namely TIR-NB-LRR (TNL), CC-NB-LRR (CNL) and RPW8-NB-LRR (RNL) (Andolfo et al. 2019, 2020). Recent studies demonstrated that NLRs can specifically detect pathogens using integrated domains (IDs) by monitoring the manipulation of host targets (Ortiz et al. 2017).

The number of NLRs varies dramatically between different plant genomes. Some crops such as hot-pepper, barrel-clover, apple and wheat showed approximately 1000 NLRs (Andolfo et al. 2020). Diversely, the Cucurbitaceae family shows a lower number of NLR genes per lineage than other species (Sun et al. 2017; Andolfo et al. 2020). All genome‐wide analyses showed that a large fraction of NLRs is arranged in clusters (Di Donato et al. 2017). NLR gene cluster organization is very ancient, the first gene cluster involved 5 TNL genes and was identified in a green alga (Andolfo et al. 2019). The gene duplications have played an important role in the evolutionary history of the R-gene family, contributing to the diversification of the defense arsenal. Until now, the NLR repertory was described in few Cucurbitaceae sequenced genomes such as cucumber, melon, watermelon, zucchini and pumpkin (Garcia-Mas et al. 2012; Lin et al. 2013; Andolfo et al. 2017; Sun et al. 2017).

Recently, a set of *Cucurbita* genomic tools has become available for breeders and researchers, including de novo genome assemblies, transcriptomes, high-quality saturated maps, TILLING platforms and genome resequencing (Sun et al. 2017; Montero-Pau et al. 2018; Barrera-Redondo et al. 2019; Xanthopoulou et al. 2019). RNA sequencing is becoming increasingly a frequent technology orienting and driving the basic genetic research (Andolfo et al. 2014a; D’Esposito et al. 2019). Transcriptome sequencing encompasses a wide variety of applications from the prediction of protein-encoding gene function to the monitoring of dynamic gene expression regulation (Wang et al. 2009).

To date, several high-quality *Cucurbita* transcriptomes have been explored, most of them attributable to *C. pepo* cultivars (Blanca et al. 2011; Andolfo et al. 2017; Montero-Pau et al. 2018). However, still little is known about the *Cucurbita* species genetic diversity and even less has been done to explore their resistance gene arsenals. The availability of transcriptomic resources in *Cucurbita* provides an opportunity to conduct the first study on the evolutionary dynamics of the NLR-encoding gene family for a representative group of *Cucurbita* species. To this end, we used 40 transcriptomes and 5 genomes belonging to 12 *Cucurbita* species carrying out the NLR gene family annotation, characterization and diversification.

## Materials and methods

### Dataset

To investigate the NLR gene family evolution in the *Cucurbita* genus, we used 5 genomes [*C. argyrosperma* subsp.* argyrosperma* (*C. argyrosperma* hereafter), *C. argyrosperma* subsp. *sororia* (*C. sororia* hereafter)*, C. maxima*, *C. moschata* and *C. pepo*] (Suppl. Table S1 and S2) (Sun et al. 2017; Montero-Pau et al. 2018; Barrera-Redondo et al. 2019, 2021) and 40 transcriptomes [5* C. argyrosperma*, 2 *C. cordata,* 3 *C. ecuadorensis,* 2 *C. ficifolia,* 3 *C. foetidissima,* 4 *C. lundelliana,* 4 *C. maxima,* 3 *C. moschata,* 3 *C. okeechobensis,* 3 *C. pedatifolia*, 7 *C. pepo*, and 1 *C. scabridifolia* accessions] from twelve* Cucurbita* species. The RNA-seq data of 40 analyzed transcriptomes were downloaded from supplementary data published by Montero-Pau et al. (2018). The genomic data were retrieved from CuGenDB (Zheng et al. 2019). For comparative purposes, we also used 96 well-characterized reference R-genes from 25 plant species retrieved from PRGdb (Osuna-Cruz et al. 2018), and 1 out-group gene with a nucleotide-binding (NB) domain, the human *Apaf1* (Apoptotic Protease Activating Factor 1) gene (Suppl. Table S3).

### Identification of NLR-domain encoding genes

**Fig. 1:**
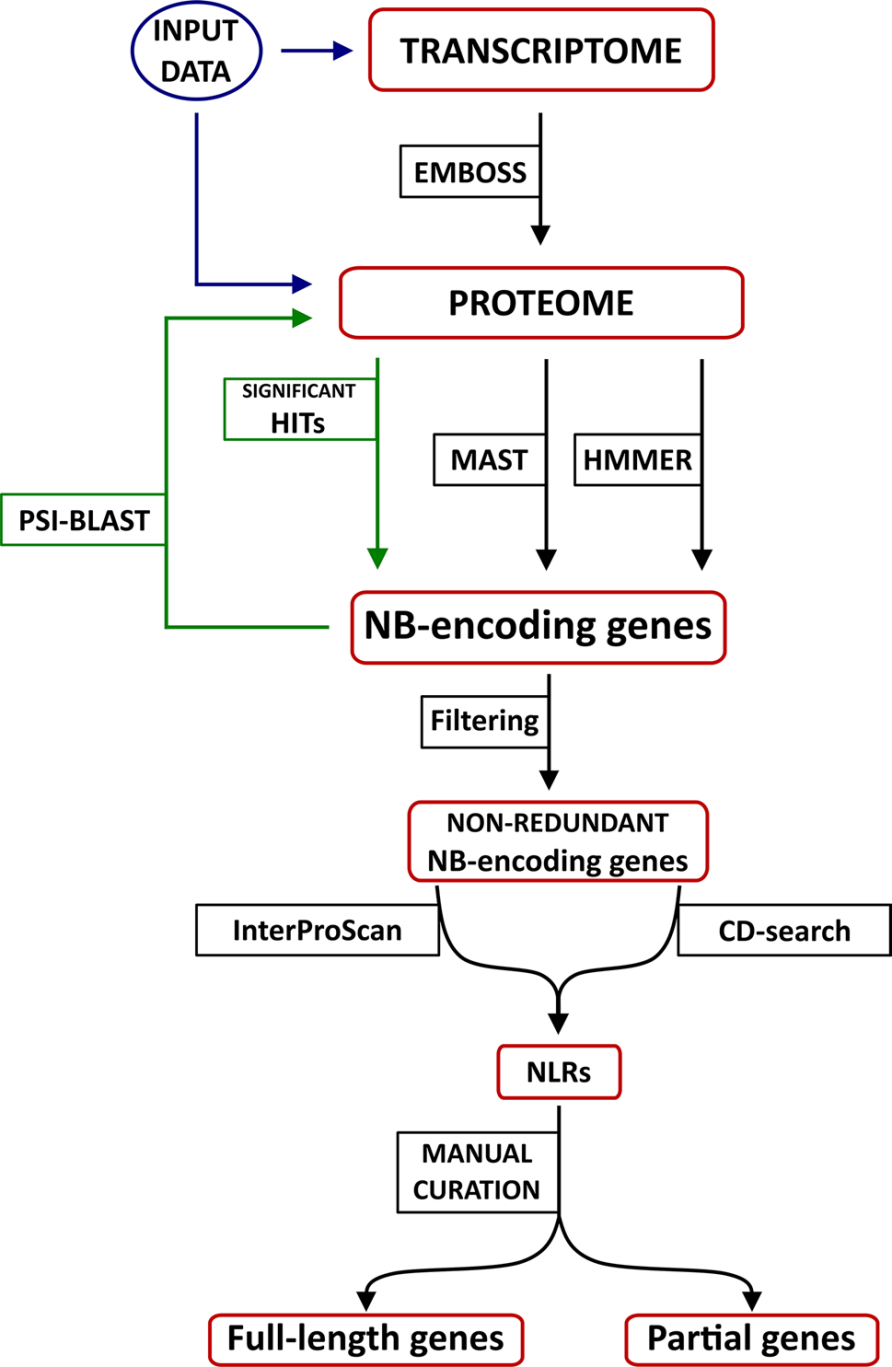
A step-by-step workflow for NLR gene annotation. The pipeline was designed to use transcript and protein sequences to detect conserved domains found in well-characterized plant R-genes (InterProScan and CD-search software) by integrating results generated from motifs-/domain-based search (MAST and HMMER software) combined with a data-mining approach through a reiterative process (PSI-BLAST)

A total of 1,990,997 transcripts and 151,141 protein sequences were analyzed to identify and annotate the *Cucurbita* expressed-NLR (eNLR hereafter) genes in 40 de novo assembled transcriptomes and 5 *Cucurbita* genome assemblies, respectively. To identify the NLR-domain encoding genes, we translated the transcripts in all six possible reading frames (three in the forward direction and three in the reverse) using EMBOSS Sixpack (Madeira et al. 2019). The Multiple EM for Motif Elicitation (MEME) (Bailey et al. 2006) algorithm was used to decompose in motifs (Suppl. Table S4) the NB Pfam domain sequences (PF00931) of functionally characterized plant R proteins. The identified MEME motifs were used by Motif Alignment and Search Tool (MAST) (Bailey et al. 2006) to identify the NLR-domain encoding genes in our dataset. The *Cucurbita* proteomes were also scanned with the hidden Markov model (HMM) of the nucleotide-binding (NB Pfam ID: PF00931) using HMMER v3.0 (Finn et al. 2011). The analysis was carried out using the default cutoff value for statistical confidence. To detect additional *Cucurbita* eNLR/NLR genes that have escaped by the foregoing annotation, a reiterative process of gene identification was implemented. The annotated *Cucurbita* NB-encoding genes were used as queries against the 45 *Cucurbita* proteomes using a PSI-BLAST search (E-value cutoff of 1e-10). The protein domain architecture of identified *Cucurbita* eNLR/NLR proteins was annotated using IntetProScan v5 (Jones et al. 2014) and Conserved Domain Search (CD-Search) (Marchler-Bauer and Bryant 2004) with default parameters. Finally, a manual curation process was included in our annotation pipeline to discover the genes encoding single or partial NLR domains (Fig. 1). *Cucurbita* NLR sequences in FASTA format can be accessed at FIGSHARE with the following link (https://figshare.com/s/b2808ef8f5a29a8a522d). Users can download and use the data freely for research purposes only with acknowledgment to us and quoting this paper as a reference to the data.

### Multiple sequence alignments and phylogenetic analysis

MAFFT (Multiple Alignment using Fast Fourier Transform) v6.814b was used to align the NB Pfam domain (PF00931) of both eNLR-/NLR-encoding genes using the L-INS-i algorithm (Katoh et al. 2002). Sequences with less than 50% of the full-length NB Pfam domain were excluded. Evolutionary analyses were conducted using MEGA 7 (Kumar et al. 2016). The phylogenetic relationships of *Cucurbita* NLR proteins were inferred using the maximum likelihood method based on the JTT model (Jones et al. 1992). The model with the lowest BIC score (Bayesian Information Criterion) was considered to describe the substitution pattern the best. The bootstrap consensus tree inferred from 100 replicates was taken to represent the evolutionary history of the analyzed sequences. The trees were drawn to scale, with branch lengths measured in the number of substitutions per site. Finally, the human *Apaf1* gene was used to reroot the phylogenetic trees, because the split of plant and animal kingdoms predates the origin of NLR gene family groups (Andolfo et al. 2020).

### Estimation of selection pressure and evolutionary divergence

Selective pressure acting on the *Cucurbita* NLRs was investigated by determining the nonsynonymous to synonymous nucleotide substitution (dN/dS) indicated as ω. Tests were conducted to estimate the evolution of each codon: positive (*ω* > 1); neutral (*ω* = 0); and negative (*ω* < 1). All positions with less than 80% site coverage were eliminated. All the NLR-coding DNA sequences were aligned using ClustalW v1.74 (Larkin et al. 2007). To depict the proportion of sites under selection, the evolutionary fingerprint analysis was carried out using the SLAC algorithm implemented in the Datamonkey server (Delport et al. 2010). The evolutionary divergence (*π*) was estimated as the number of base substitutions per site from averaging over all sequence pairs. Analyses were conducted using the Maximum Composite Likelihood model (Tamura et al. 2004). All positions containing gaps and missing data were eliminated. Evolutionary analyses were conducted in MEGA7 (Kumar et al. 2016).

### Changes in NLR gene family size

The changes in *Cucurbita* NLR gene family sizes, across the dated phylogeny (Barrera-Redondo et al. 2019), were assessed using CAFE v4.2.1 with default settings (De Bie et al. 2006). To analyze significant expansion/contraction events of NLR orthogroups throughout the *Cucurbita* tree, we used the *λ* value (0.01397) estimated by Barrera-Redondo et al. (2019), which takes into account the genome assembly and annotation errors for all analyzed genomes (Han et al. 2013). The average expansion size indicates the mean number of genes gained or lost per orthogroup. *C. sororia* NLR gene family was excluded from evolutionary change analyses because our ortholog dataset was inadequate to correctly estimate a ʎ value including *C. sororia*.

### Validation of eNLRs’ expression by qPCR

NLRs’ expression was validated by quantitative PCR (qPCR) in nine different transcriptomes of the analyzed *Cucurbita* accessions. Three seeds of each accession were disinfected with 10% sodium hypochlorite, rinsed with distilled water and then germinated by incubation over cotton in Petri plates at 37 °C for 48 h. Seedlings were grown in a climatic chamber at 26 °C and 60% of relative humidity, under a photoperiod of 16 h day/8 h night. Leaf tissue of plants at the third true-leaves stage was sampled and immediately frozen in liquid nitrogen. Total RNA was extracted using Extrazol (EM30) (Blirt, Gdańsk, Poland) and quantified using a NanoDrop 1000 spectrophotometer (Thermo Scientific™, San José, CA, USA). Aliquots of 1.5 µg were treated with Perfecta DNase I (Quanta Biosciences, Gaithersburg, MD, USA), and the first strands of cDNA were reverse transcribed with RevertAid synthesis Kit (Thermo Scientific™) and Oligo(dT) primer.

Four NLRs were amplified per accession using 39 primer pairs (Suppl. Table S5), which were designed by Primer 3Plus software (Untergasser et al. 2007). qPCR reactions were performed on a LightCycler^®^ 480 System (Roche Diagnostics, Mannheim, Germany), conducting two technical replicates and using 7.5 μL of the ready-to-use FastStart Essential DNA Green Master mix (Roche Diagnostics), 1.5 μL (1 µM) of each primer and H_2_O to a final volume of 15 μL. Reactions were conducted under the following conditions: 95 °C for 5 min, 40 cycles of 95 °C for 15 s, 60 °C for 30 s, and 72 °C for 15 s, ending up with a melting curve step (60 °C–95 °C) to check the specificity of each fragment. Relative expression of the NLRs amplified was calculated following the 2^−ΔCt^ method, described by Schmittgen and Livak (2008). The ubiquitin fusion protein (UFP) gene was used as an endogenous calibrator (Obrero et al. 2011).

## Results

### NLR-resistance gene annotation in *Cucurbita* species

A total of 1603 eNLR and 256 NLR genes were identified in 40 transcriptomes and 5 genomes, respectively, using a hybrid strategy of gene annotation (Table 1 and 2; Suppl. Tables S6 and S7). A fine R-gene motif-/domain-based search (Suppl. Table S4) was combined with a data-mining approach through a reiterative process (Fig. 1). In the first step, approximately 500 NB-encoding genes were identified, using MAST and HMMER software for sequence similarity searches. The resulting NB-encoding genes were annotated and classified based on the features of their N-terminal and C-terminal protein structures. Finally, all NLR-domain encoding sequences were used as queries against the entire 45 *Cucurbita* assemblies to check for additional genes that had not been previously identified (Fig. 1).

**Table 1:** The *Cucurbita* spp. expressed-NLR gene repertoires

Species	Accession number	Geographic origin	NRL-encoding genes (n.)^a^
*C. argyrosperma*	PI-438547	Belize	64
	PI-512114	Nicaragua	38
	PI-512115	Guatemala	45
	PI-451712	Mexico	75
	PI-202079	Mexico	46
*C. cordata*	GRIF-9445	Mexico	17
	PI-653839	Mexico	35
*C. ecuadorensis*	PI-432441	Ecuador	26
	PI-432443	Ecuador	16
	GRIF-9446	Ecuador	36
*C. ficifolia*	CATIE-16038	Guatemala	44
	CATIE-16575	Guatemala	48
*C. foetidissima*	PI-442197	Mexico	41
	PI-532350	Mexico	34
	PI-442201	Mexico	51
*C. lundelliana*	PI-438542	Belize	46
	PI-540898	Honduras	52
	PI-532357	Mexico	66
	PI-636138	Belize	60
*C. maxima*	VIR-3202	Chile	32
	BGV004558	Argentina	34
	UPV035142	Angola	26
	PI-458653	Argentina	21
*C. moschata*	PI-498429	Colombia	35
	NIG-LOCAL	Nigeria	64
	PI-653064	Nigeria	42
*C. okeechobensis*	PI-532363	Mexico	33
	PI-512105	Mexico	28
	PI-512106	Mexico	16
*C. pedatifolia*	PI-442341	Mexico	42
	PI-442290	Mexico	73
	PI-540737	Mexico	33
*C. pepo*	CATIE-18887	Mexico	41
	CATIE-11368	Guatemala	34
	BGV004370	Spain	10
	BGV005380	Spain	10
	PI-615111	USA	28
	PI-532354	Mexico	71
	PI-614701	Mexico	37
*C. scabridifolia*	PI-532392	Mexico	53

^a^Numbers of genes that encode domains similar to plant R proteins as identified in this study

**Table 2:** The full NLR gene complements of five *Cucurbita* genome assemblies

Protein domains^a^	*C. argyrosperma*	*C. maxima*	*C. moschata*	*C. pepo*	*C. sororia*
Full-length					
CC-NB-LRR	3	14	28	5	9
TIR-NB-LRR	4	5	14	1	10
RPW8-NB-LRR	5	5	5	6	5
Total full-length^b^	**12**	**24**	**47**	**12**	**24**
Partial					
CC-NB	3	3	4	–	2
CC-LRR	–	–	2	2	–
TIR-NB	1	3	5	3	5
TIR-LRR	–	–	–	1	–
NB	1	5	4	2	4
TIR	6	10	9	5	10
LRR	2	7	15	14	4
RPW8	2	1	1	2	2
Total partial^b^	**15**	**29**	**40**	**29**	**27**
Total^c^	**27**	**53**	**87**	**41**	**51**

^a^*CC* coiled coil; *LRR* leucine-rich repeat; *NB* nucleotide binding; *RPW8* resistance to powdery mildew 8; *TIR* toll/interleukin receptor

^b^Subtotals of partial and full-length NLR genes

^c^Amount of annotated NLR genes

**Fig. 2:**
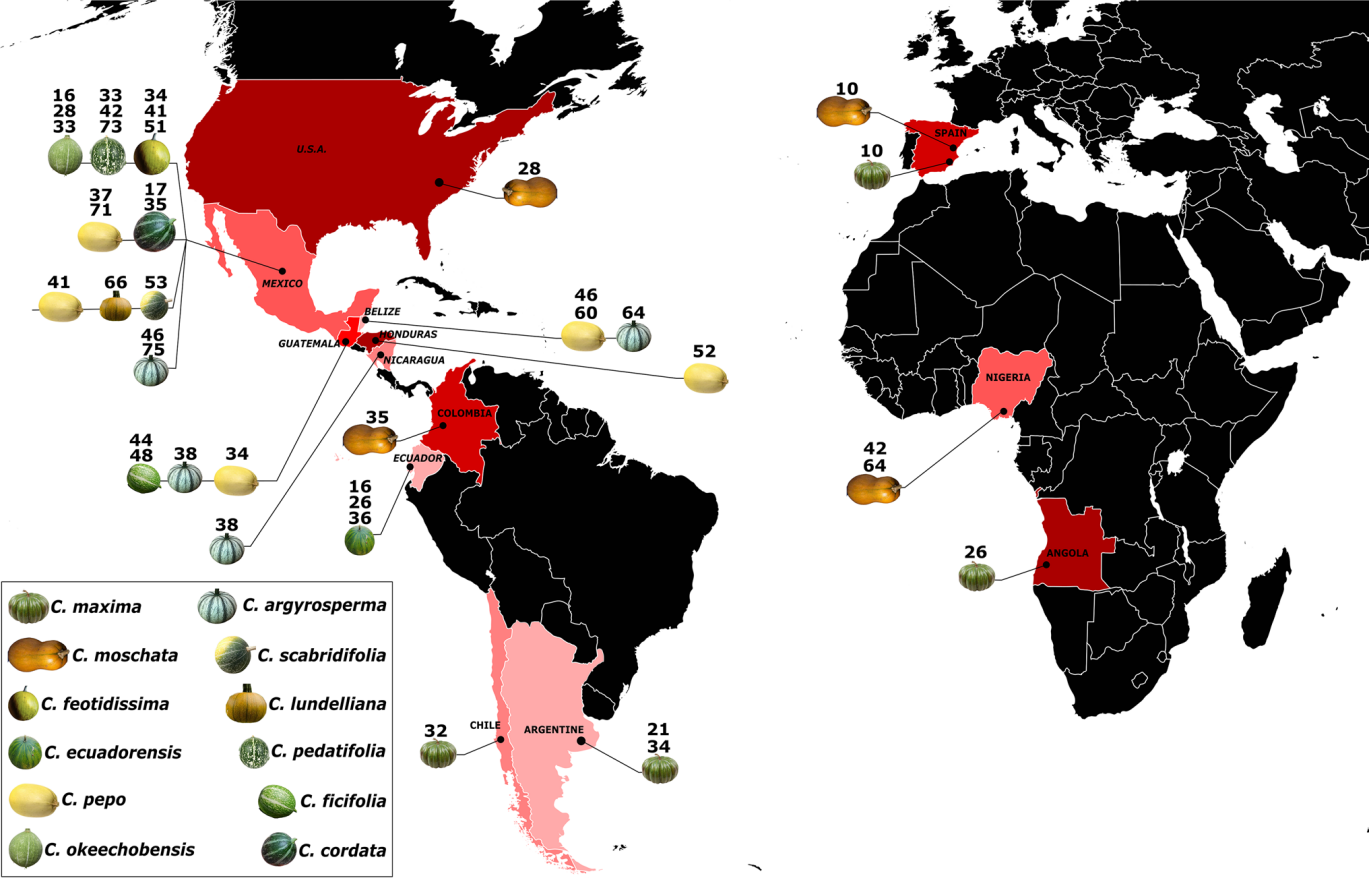
Geographical origins of 40 *Cucurbita* transcriptomes and the relative sizes of NLR gene families. Peponid pictures at the end of the links indicate the *Cucurbita* specie. Total NLR number is indicated on the top of each fruit cartoon. For multiple accessions of the same species, the NLR family sizes are indicated on the same peponid image

The full NLR complements ranged widely, from 87 to 25 members in *C. moschata* and C. *argyrosperma*, respectively (Table 2), a number substantially lower than in other plant species (Di Donato et al. 2017; Barchi et al. 2019; Andolfo et al. 2020). Interestingly, *C. sororia* exhibited 51 NLR genes a number significantly (*P* < 0.05) higher than its domesticated counterpart, *C. argyrosperma*. Likewise, the analyzed transcriptomes, deriving from 40 *Cucurbita* accessions, come from 6 geographic regions of the world (Fig. 2), exhibited an average of 40 eNLR/NLR genes. Currently, *C. pedatifolia* (from Mexico) holds the highest number of eNLRs (73) among all *Cucurbita* spp., while, the lower number of eNLRs (10) was recorded in the 2 Spanish *C. pepo* accessions (Table 1). Differences in the total number of eNLRs within species were found (e.g., *C. foetidissima; C. argyrosperma; C. okeechobensis*) (Table 1). Moreover, only 4% (58 out of 1603) of annotated eNLRs encode the minimal domain structure (NB and LRR) necessary for a full-length NLR-encoding gene (Table 1). This finding was unlikely to reflect the true structure of 40 *Cucurbita*-resistance (*R*) gene families and might be due to the fragmented nature of the 40 transcriptomes since more than 70% (1162) of the eNLR proteins are encoded from very short transcripts (length < 700 bp). At least one full-length eNLR gene for each of the main R protein classes (CNL, RNL and TNL) was annotated in each of the analyzed *Cucurbita* sp. (Table 1). Generally, the NLRs belonging to the CNL class outnumbered those of the TNL class of one-third (Table 2). Interestingly, the full-length RNLs were especially numerous in the *Cucurbita* genomes (Table 2 and Suppl. Fig. S1).

### NLRs genome organization and protein domain arrangement

The genome-wide distribution of NLRs (Table 2), based on the chromosome size, was significantly non-random in *C. maxima* (*χ*^2^ = 101, *P* < 0.01) and *C. moschata* (*χ*^2^ = 150, *P* < 0.01). Diversely, in *C. argyrosperma* and *C. pepo* genomes, the NLRs were casually dispersed (*C. argyrosperma*: *χ*^2^ = 22, *P* > 0.05, C. pepo: *χ*^2^ = 26, *P* > 0.05 and *C. sororia* (*χ*^2^ = 68, *P* < 0.01) and no gene cluster was identified.

The chromosomes 5, 6 and 14 of *C. maxima* and *C. moschata* genomes are particularly enriched of NLRs (over 50% of total genes), while NLRs are absent on chromosomes 8, 12 and 19. The 37% of *C. sororia* NLRs were located on chromosomes 5 and 9. Based on Richly et al. (2002) definition of a gene cluster (a spatial arrangement of NLRs by a gap of no more than 8 non-NLRs), we found that 51 (out of 87) and 12 (out of 53) NLRs resided in clusters in *C. maxima* and *C. moschata*, respectively. The largest cluster was located on chromosome 5 and includes 8 and 12 NLRs in *C. maxima* and *C. moschata*, respectively. *C. sororia* exhibited the biggest cluster on chromosome 9 including 8 TNLs. The 5 *Cucurbita* genomes hold 21 pairs and 6 triplets. Therefore, 77%, 55%, 26%, 22% and 65% of NLRs resided either in a gene cluster or in an array of two or three genes in *C. moschata*, *C. maxima*, *C. argyrosperma*, *C. pepo* and *C. sororia*, respectively (Table 3).

**Table 3:** NLR gene clusters found in the full-assembled *Cucurbita* genomes

Species^a^	Singletons	Grouped NLRs			Average total no of NLRs in cluster	Total no of grouped NLRs (%)
		Pairs	Triplets	Clusters		
*C. argyrosperma*	20	2	1	0	–	7 (26)
*C. maxima*	24	7	1	2	6	29 (55)
*C. moschata*	20	5	2	9	5.7	67 (77)
*C. pepo*	32	3	1	0	–	9 (22)

^a^*C. sororia* was omitted since its genome was partially assembled

A substantial domain reshuffling was observed in the *Cucurbita* R-gene family, several NLRs showed atypical protein domain assemblies (Suppl. Fig. S2). A total of 7 IDs (Metallo-dependent hydrolase domain; SNARE fusion complex domain; cytochrome b5-like heme/steroid binding domain; pectin methyl-esterase inhibitor domain; Pex2-Pex12 domain; kinase domain; zinc finger-like domain) were identified in the *Cucurbita* NLR family. Several *Cucurbita* NLRs with transmembrane (TM) signatures have been identified, mostly related to the TNL class (Suppl. Fig. S2).

### Phylogenetic analysis of *Cucurbita* NLR genes

**Fig. 3:**
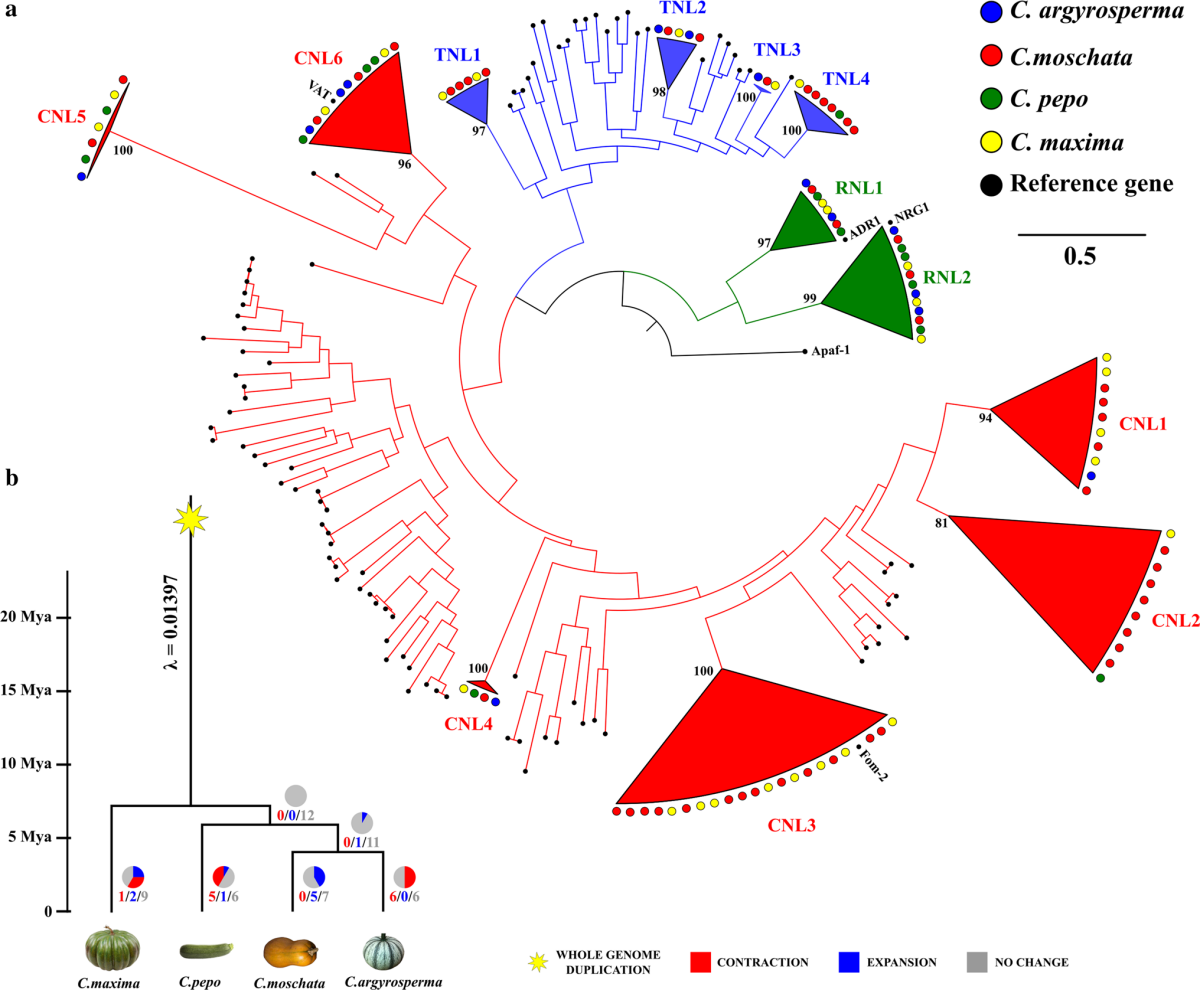
Evolutionary history of *Cucurbita* NLR gene family. **a** Phylogenetic tree of *Cucurbita* NLR gene family identified in four genome assemblies. Clades are collapsed based on a bootstrap value over 80 and numerated. **b** NLR gene family expansions and contractions along dated phylogeny of the *Cucurbita* genus. The tart charts and the numbers at every branch of the tree indicate whether a gene family expanded (blue), contracted (red), or remained the same size (gray). The gene birth/death rate (*λ*) in the most recent common ancestor of the phylogeny and the whole-genome duplication (yellow star) in *Cucurbita* sp. were indicated

The evolutionary events, driving the *Cucurbita* NLR gene family, were inferred by analyzing the full NB domains extracted from each NLR gene annotated in four *Cucurbita* genome assemblies (Table 2). For comparative purposes, we included 96 well-characterized plant R-genes (Osuna-Cruz et al. 2018) and 1 out-group gene with an NB domain, the human Apaf-1 (Suppl. Table S3). A total of 106 NLRs were aligned together with the same reference R dataset (Fig. 3a; Suppl. Fig. S3; Suppl. Table S3). The analyzed NB Pfam domains collapsed in 12 robust clades (bootstrap index > 80), of which only 5 (CNL4, CNL5, CNL6, RNL1 and RNL2) included at least 1 gene belonging to each analyzed genome (Fig. 3a; Suppl. Fig. S3). Moreover, we found the direct orthologs only for *Fom-2*, *Vat*, *ADR1* and *NRG1* reference genes (Suppl. Fig. S3). The *C. pepo* and *C. argyrosperma* proteomes no-included *Fom-2* homologs, while in *C. maxima* (8) and *C moschata* (13) were especially numerous (CNL3 clade in Suppl. Fig. S3). The BLASTn search of melon *Fom-2* coding DNA sequence against the *Cucurbita* genome assemblies uncovered 64 and 76 significant exon-matches (*E*-value < 1e-6) in *C. pepo* and *C. argyrosperma*, respectively (Suppl. Table S8). Surprisingly, the clades CNL3 and TNL1 grouped ~ 35% of *C. maxima* and *C moschata* NLR complements. Overall, the difference in NLR arsenals is due to the gain/loss of specific gene subfamilies across *Cucurbita* species.

**Fig. 4:**
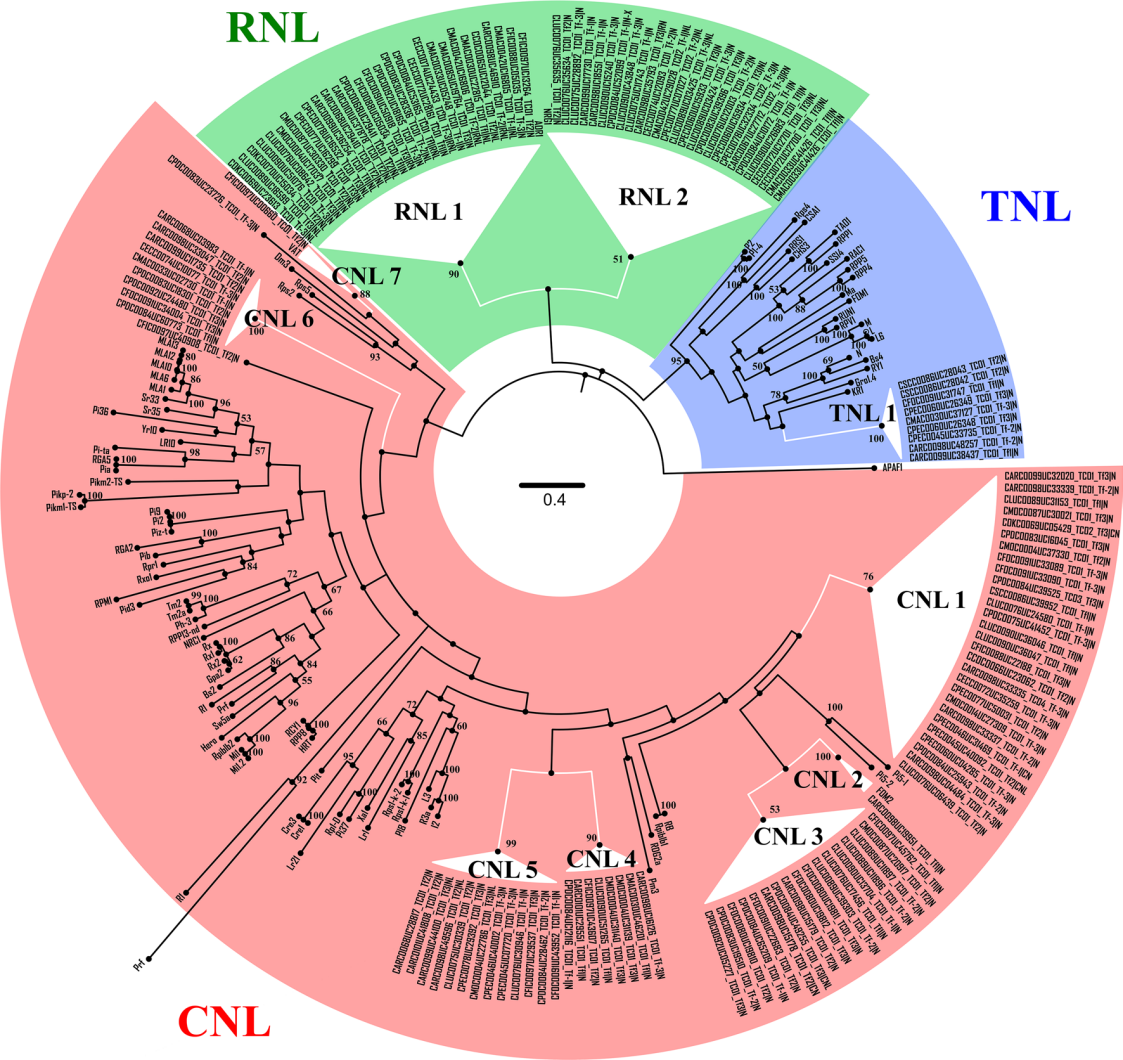
Molecular phylogenetic analysis of *Cucurbita* expressed-NLR genes. Evolutionary history inferred using the maximum likelihood method based on the JTT model. The tree is drawn to scale, with branch lengths measured in the number of substitutions per site. Clades are collapsed based on a bootstrap value over 50 and numerated. The TNL, RNL and CNL groups are drawn with blue, green and red backgrounds, respectively

To integrate and validate the above-mentioned findings, we performed a phylogenetic analysis on eNLR gene identified in 40 *Cucurbita* transcriptomes. A total of 243 amino acid sequences were aligned and collapsed into three groups (TNL in blue, RNL in green and CNL in red) supported by bootstrap values ≥ 50 (Fig. 4). Within the three groups, ten clades (CNL1 to 7, RNL1 and 2 and TNL1) were defined. Twenty-eight *Cucurbita* NLRs belonging to all domesticated species, except *C. maxima*, as well as to all wild types were harbored into the clade CNL1. They were identified in *C. pepo* accessions of the three subspecies: subsp. *pepo* pumpkins from Central America, subsp. *ovifera* acorn from USA and wild subsp. *fraterna* from Mexico, but not in the Spanish subsp. *pepo* accessions. Clade CNL2 comprises a CNL gene (CARCO098UC19951) annotated in the Indian accession *C. argyrosperma* PI-451712, the one with the highest NLR number. This gene showed very high homology to the melon gene *Fom-2*, which confers resistance to *Fusarium oxysporum* f.sp. *melonis* races 0 and 1 (Joobeur et al. 2004). Seventeen similar sequences were identified in 6 different *Cucurbita* spp. (Central America domesticate *C. argyrosperma*, *C. ficifolia*, *C. pedatifolia*, *C. lundelliana* and *C. foetidissima* and a Nigerian *C. moschata* accession) and are grouped in clade CNL3. It is interesting to note that clade CNL4 and CNL5 share a common ancestor (supported by 83% bootstrap indexes). CNL4 includes eight members from three domesticates, including *C. maxima* but not *C. pepo*, and two wild species, whereas Clade CNL5 holds genes from the economically important *C. pepo* species. This clade also includes genes from *C. argyrosperma*, *C. ficifolia* and *C. moschata* and from the wild types *C. pedatifolia*, *C. lundelliana* and *C. foetidissima*, all from Central America. The clade CNL6 contains nine CNLs. The direct ortholog (CFIC0097UC00660 identified in *C. ficifolia*) of *Vat* (virus aphid transmission) gene (Dogimont et al. 2014), conferring resistance to both *A. gossypii* and the viruses, falls into clade CNL7 (Fig. 4). Distinct from the canonical CNL genes are the RNL homologs divided into two robust clades (RNL1 and RNL2) (Fig. 4). RNL sequences are located on an ancestral position between TNL and CNL groups, and harbor homologs to the *Arabidopsis ADR1* (Activated Disease Resistance 1) gene and *Nicotiana benthamiana NRG1* (N requirement gene 1) (Peart et al. 2005; Bonardi et al. 2017). Overall, 27 and 21 *Cucurbita* RNL genes were grouped into the RNL1 (ADR1-homologs) and RNL2 (NRG1-homologs) clades, respectively. At least one *ADR1*-homolog was identified in each of the analyzed *Cucurbita* sp., while not all showed their *NRG1*-homolog. Finally, TNL1 harbors nine genes from eight *Cucurbita* species.

### *Cucurbita* NLR gene expansion and contraction events

The wide range of NLR family sizes and diversification prompted us to investigate the gene birth and death processes that occurred in the *Cucurbita* evolutionary history (Table 2). To assess the expansions and contractions of NLR family along the *Cucurbita* phylogenetic tree, we have used the orthology relationships indicated in the NLR phylogenetic tree (Fig. 3a; Suppl. Fig. S3). We employed the global gene birth–death rate (*λ*) reported by Barrera-Redondo et al. (2019), to estimate the NLR gene gain–loss across *Cucurbita* phylogeny (Fig. 3b). Five NLR orthogroups (CNL1, CNL2, CNL3, TNL1 and TNL4 clades of Fig. 4a) significantly changed (*P* < 0.01) into the *Cucurbita* genus. Moreover, the TNL2 clade showed a lower, but significant (*P* < 0.05) level of change.

The average expansion size of *C. moschata* NLR family was 2, while *C. argyrosperma* (average expansion size = − 1) and *C. pepo* (average expansion size = − 0.92) showed a global contraction of NLR families. The difference in the proportion of *C. maxima* NLRs, across all orthogroups, was almost nil (average expansion size = 0.08). The branches leading to *C. maxima* and *C. moschata* exhibited the highest number of orthogroups in the rapid expansion (Fig. 3b). In particular, the *Fom-2* homologs (CNL3 clade in Fig. 3a) were significantly expanded in *C. maxima* and *C. moschata.* The differences in *Fom-2* homolog content across *Cucurbita* species were corroborated by several significant exon-matches identified into the *Cucurbita* assemblies (Suppl. Table S8). Even though few orthogroups (CNL2 and TNL4 clades in Fig. 4a) showed significantly rapid (*P* < 0.01) levels of change in the branch leading to *C. pepo*, this branch had the highest number of orthogroup (OG) changes (8 out of 12) within the entire phylogeny (Fig. 3b).

### Evaluation of selection pressure acting on *Cucurbita* NLRs

To infer the direction and degree of natural selection acting on the *Cucurbita* NLRs, we estimated the variation level between the nonsynonymous substitution (*dN*) and synonymous substitution (*dS*) values. Results of the neutrality test performed for eight (out of ten) phylogenetic clades (Fig. 3) using the SLAC method (Pond and Frost 2005) are summarized in Table 4. The selection pressure expressed as dN/dS (*ω*) values of eight NLR-coding sequence alignments greatly varies from 0.48 to 1.2. Purifying selection (*ω* < 1) was observed in TNL1, RNL1-2, and in all CNL clades excepted for CNL5 (*ω* = 1.2). A consistent number (until 49) of codons under negative selection were identified for each clade (Table 4). A global stabilizing selection (ranging from 0.49 to 0.57) is acting against polymorphic variants in all the *Cucurbita* R-gene groups (Fig. 3), indicating the need to preserve their state (Table 4). The single codon analysis underlined 6, 6 and 1 positively selected sites in CNL1, RNL1 and RNL2 clades, respectively (Table 4). Five positively selected codon sites of ADR1-homologs (RNL1 clade in Fig. 3) are located on the NB Pfam domain (PF00931). Probably, the global protein structure of ADR1-like genes has been conserved, but the positive selection in specific sites of NB domains has been promoted to generate novel variability. The overall analysis of CNL and RNL phylogenetic groups underlined a conspicuous number of codons under positive (21 and 13 in CNL and RNL, respectively) and negative (98 and 67 in CNL and RNL, respectively) selection (Table 4).

**Table 4:** Estimation of evolutionary divergence (*π*) and nonsynonymous and synonymous substitutions mean dissimilarity (*ω* = *dN*/*dS*) for each phylogenetic clade of *Cucurbita* eNLR genes

	NLR-CDSs^A^	NLRs (*n*.)	Π	SLAC^C^		
				*ω*	PSCs (*n*.)	NSCs (*n*.)
Clade	CNL1	28	0,061	0,62	6	44
	CNL3	17	0,039	0,53	0	12
	CNL4	8	0,114	0,9	0	3
	CNL5	13	0,075	1,2	0	0
	CNL6	9	0,151	0,94	0	0
	RNL1	29	0,075	0,51	6	31
	RNL2	27	0,159	0,48	1	49
	TNL1	9	0,035	0,51	0	7
Group^B^	CNL	77	0,048	0,56	21	98
	RNL	56	0,094	0,49	13	67

^A^Datasets of analyzed NLR-coding DNA sequences (eNLR-CDSs)

^B^Clade TNL1 coincide with TNL group

^C^Number of positive (PSCs: positively selected codon sites) and negative (NSCs: negatively selected codon sites) codons statistically significant in coding DNA sequences

Finally, the evolutionary divergence (π values in Table 4), calculated as mean base substitution per site over all sequence pairs, oscillated from 0.035 (TNL1 clade) to 0.159 (RNL2 clade). The low level of nucleotide diversity within eight clades indicated a limited genetic variation between phylogenetic homologs. While comparing the three phylogenetic groups (Fig. 3), the *Cucurbita* RNLs exhibited the highest nucleotide variability (*π* = 0.094). Although, the ADR1-/NRG1-homologs are conserved in *Cucurbita* spp., with respect to well-characterized plant R-genes, they exhibited the widest range of sequence diversity in coding regions.

### Molecular validation of *Cucurbita* expressed-NLR genes

To verify that the de novo assembled eNLRs were present in *Cucurbita* transcriptomes a molecular analysis was carried out. A total of 36 eNLR genes selected in 9 *Cucurbita* accessions were molecularly validated by qPCR. Specific fragments were amplified to all the genes tested, verifying their transcription in the assayed accessions (Supp. Fig. S4). Relative expression values (2^−ΔCt^) were consistent with the level of transcripts detected by sequencing, which demonstrates the veracity of the eNLR gene expression in the studied transcriptomes.

## Discussion

A reiterative process implemented in an ad hoc R-gene annotation approach was used to identify the genes that encode domains similar to plant R proteins in 45 *Cucurbita *de novo assemblies (including both transcriptomes and genomes). The NLR gene families show a wide range variations into analyzed *Cucurbita* species. In our study, NLR gene families ranged from 87 to 27 members in *C. moschata* and *C. argyrosperma*, respectively. On average, the number of NLR loci in the Cucurbitaceae family is significantly lower than in other plant families (Lin et al. 2013; Andolfo et al. 2020). Recently, Guo et al. (2020) identified whole-genome duplication (WGD) events in the Cucurbiteae, which have surely contributed to their evolutionary diversification. The polyploidizations events provide abundant duplicated genes (Soltis and Soltis 2016), but only a small fraction is maintained (Zhang et al. 2012). Probably, most of the ancient duplicated NLRs were lost before the cucurbit speciation.

On average, a slightly higher number of CNLs compared to TNLs was identified in most *Cucurbita* assemblies. A similar CNL\TNL ratio was found in Solanaceae (Di Donato et al. 2017; Andolfo et al. 2021), while the Brassicaceae and Fabaceae contain from twofold to sixfold more TNL than CNL genes (Guo et al. 2011; Kang et al. 2012). Surprisingly, the *Cucurbita* RNL (also known as helper) genes varied from ~ 18 to ~ 6% of the full NLR gene complement (Suppl. Fig. S1). It is a conspicuous fraction compared to that of many other Angiosperms (Andolfo et al. 2020). The helpers are essential in defense-related gene regulatory networks for increasing the robustness of the innate immune system (Wu et al. 2018). *Cucurbita* NLR gene complements include several genes carrying integrated domains (IDs), which act as “baits” enabling pathogen recognition (Sarris et al. 2016). NLR-ID proteins for functioning may require an appropriate helper or NLR partner and when transferred as a unit between plant families confer resistance to diverse pathogens (Nguyen et al. 2021).

The speed of adaptation to changing pathogen populations of plant species is crucial for their survival, therefore, the availability of a varied NLR defense arsenal may be useful (Andolfo and Ercolano 2015; Di Donato et al. 2017). Tandem duplication, exon shuffling, gene gains and losses have a decisive role in NLR genes diversification of *Cucurbita* genus (Lin et al. 2013; Andolfo et al. 2017). *C. maxima* and *C. moschata* preferentially tend to organize theirs defense arsenals in clusters, and most of them are derived from recent gene duplications. By contrast, *C. pepo* and *C. argyrosperma* genomes showed a random genome-wide NLR organization without clear gene clustering events. The cluster formation may facilitate the NLR gene variability by sequence recombination (Andolfo et al. 2013; Joshi and Nayak 2013), but the fitness cost can prevent R locus fixation. Therefore, NLR expansion in a genome could be limited to balance the energetic cost for R-gene transcription, translation and regulation (Tian et al. 2003). Comparative analyses of zucchini, cucumber, melon and watermelon reveal that the NLR lineages present in Cucurbitaceae family predate speciation (Harris et al. 2009; Andolfo et al. 2017; Román et al. 2020). The rare events of recent duplications influenced the expansion trajectories of cucurbits R-gene family (Wan et al. 2012). The plant species of Fabaceae and Rosaceae taxa, evolutionarily close to the cucurbits, displayed significantly larger NLR families (Jia et al. 2015), pointing out a meaningful impact of Cucurbitaceae clade divergence on the fate of its R-genes. In three Cucurbitaceae species (*C. pepo*, *C. melo* and *C. sativus*), several cucurbit-specific expansions of cell surface receptors (receptor-like kinase (RLK) and receptor-like protein (RLP) were identified, suggesting that the limited number of NLRs is complemented from the expansions of RLKs and RLPs (Andolfo et al. 2017).

In our dataset, the variation by multiple fold size of the eNLR family has been found not only within the genus but also among accessions belonging to the same species. The eNLR genes found in this study in some species resulted lower than *Cucurbita* repertories already reported (Andolfo et al. 2017; Román et al. 2020). The NLRs are biotic-stress responsive genes, usually, constitutively expressed at a low level in the plants (McHale et al. 2006). All the eNLR genes molecularly tested were shown to be transcribed. The transcriptome-wide atlas of eNLR sequences was produced from RNA sequencing data of healthy leaf tissues of *Cucurbita* species and slight differences in eNLR expression levels may influence their annotation. In addition, the fragmented nature of the 40 transcriptomes may reduce the correct identification of eNLR genes. It is well known that the duplicated genes can collapse in assemblies (Bayer et al. 2018) and automated annotation systems produced approximately 30% of errors in NLR identification (Meyers et al. 2003; Andolfo et al. 2014b). In our study, only 4% of automated annotated genes showed full-length NLR structure. Therefore, we integrated a manual curation of data that enabled the discovery of NLR genes encoding single or partial NLR domains. Moreover, the R-genes tend to be masked by the genome annotation pipelines using public databases for transposable elements (Bayer et al. 2018). Indeed, our BLAST search identified several significant exon-matches for Fom-2 into the *C. pepo* and *C. argyrosperma* genomic regions devoid of predicted gene models. The observed variation of eNLR family sizes could be also accounted for R-genes losses in domesticated *Cucurbita* accessions. In addition, the differences between the BLASTn matches and the annotated gene models for Fom-2 could be the result of pseudogenization processes during domestication. This is consistent with the fact that 4 (out of 5) of analyzed genomes belong to domesticated taxa. Our R-gene annotation underlined a significant contraction of NLR gene family in *C. argyrosperma* compared to its wild relative *C. sororia* (Barrera-Redondo et al. 2021). The selective pressures imposed during domestication actively purge deleterious alleles, such as those involved in the defense mechanisms (Moreira et al. 2018).

The reconstruction of the evolutionary history of the *Cucurbita* eNLR and NLR gene family allowed the identification of three main branches (TNL, CNL and RNL). Comparing our *Cucurbita* NLR repertory with well-characterized plant R-genes, we noticed that the *Cucurbita* eNLRs collapsed in clades without reference R-genes. Six individual large clades (CNL1, CNL3-6 and TNL1) do not have similarity to any well-characterized reference R-gene, and might thus be potential sources of resistance to specific *Cucurbita* pathogen and pest elicitors (Andolfo and Ercolano 2015). Surprisingly, the drastic contraction of *Cucurbita* R family is associated to a species-specific diversification of CNL and TNL protein classes. Indeed, plant NLRs have been evolved in diverse families and subfamilies (Shao et al. 2016; Andolfo et al. 2020). Direct orthologs were identified only for *Vat* and *Fom-2,* two important CNL genes cloned in melon (Joobeur et al. 2004; Dogimont et al. 2014). Instead, the *Cucurbita* RNLs collapsed in two distinct clades together with the relative reference R-genes, ADR1 and NRG1 (Peart et al. 2005; Bonardi et al. 2017) confirming that RNLs are well conserved (Shao et al. 2016). The variations in *Cucurbita*-specific NLR subfamilies may depend on their involvement in successful stress responses during the host–pathogen interaction, resulting in their selection to the detriment of several NLR lineages. The ratio of nonsynonymous (Ka) to synonymous (Ks) nucleotide substitutions among coding DNA sequences of phylogenetic clades was below 1, except for clade CNL5. Heterogeneity of impact of selection on NLRs has been previously reported in plants (McHale et al. 2006). The NB domain of R-genes is reported to be more commonly subjected to purifying selection (Yang et al. 2006; Wan et al. 2012); the negatively selected codon sites were located on the NB-encoding region. Our results confirmed that global purifying selection act on *Cucurbita* TNL, CNL and RNL genes, but neuroses codon sites under positive/negative selection were identified.

## Conclusion

In this study, we report the first *Cucurbita*-wide annotation and characterization of NLR and eNLR genes. The IDs identification, chromosomal locations, phylogenetic relationships and species gene gains/losses’ analysis provided in this paper will contribute to the identification, isolation and characterization of R-genes. New insights into the TNL, CNL and RNL gene evolution and diversification of Cucurbitaceae family and were revealed. An important result for future applications was the reduction of R-gene family complexity from eNLR atlases, thus avoiding sequence analysis of non-expressed paralogues. The eNLR and NLR repertories identified into the 45 de novo assemblies may represent a useful resource for performing both function analysis of R-genes and genetic improvement.

### *Author contribution statement*

GA was primarily involved in experimental design, data analysis and manuscript writing. CSS planned and performed molecular experiments. CJ conceived the study and revised the manuscript. BP contributes to the draft revising of the manuscript. MRE was involved in the study design, text writing and work coordination. All the authors read and approved the final manuscript.

## Supplementary Information

Below is the link to the electronic supplementary material.

Supplementary file1 Suppl. Fig. S1 Overview of RNL gene subfamily identified in 78 seed plant genomes. Correlation plot between the number of RNL genes and relative NLRs family size (Cucurbita genomes in red and melon, watermelon and cucumber in green). (TIF 113 KB)

Supplementary file2 Suppl. Fig. S2 Schematic representation of Cucurbita NLR proteins and relative introgressed domains (IDs). MDH: Metallo-dependent hydrolase; SNARE fusion complex; Cytochrome b5-like heme/steroid binding domain; PMEI: pectin methyl-esterase inhibitor; PEX: Pex2-Pex12 domain; Kin: kinase domain; Zf: zinc finger-like domain; TM: transmebrane domain. (TIFF 208 KB)

Supplementary file3 Suppl. Fig. S3 Phylogenetic analysis of NLR genes identified in C. argyrosperma, C. maxima, C. moschata and C. pepo genome assemblies. The color of phylogenetic groups refers to Fig. 3. (TIFF 1734 KB)

Supplementary file4 Suppl. Fig. S4 Relative abundance of 36 eNLR gene transcripts annotated in 9 Cucurbita accessions. Standard error bars refer to three biological replicates. The values are reported as 2-ΔCt. UFP was used as endogenous gene. (TIFF 3161 KB)

Supplementary file5 (XLSX 58 KB)

## Abbreviations

CNL: CC-NB-LRR
LRR: Leucine-rich repeat
NB: Nucleotide-binding site
NLR: Nucleotide-binding site and leucine-rich repeat
RNL: RPW8-NB-LRR
R-gene: Resistance gene
TNL: TIR-NB-LRR

## References

[CR1] Andolfo G, Ercolano MR (2015). Plant innate immunity multicomponent model. Front Plant Sci.

[CR2] Andolfo G, Sanseverino W, Rombauts S (2013). Overview of tomato (*Solanum lycopersicum*) candidate pathogen recognition genes reveals important *Solanum* R locus dynamics. New Phytol.

[CR3] Andolfo G, Ferriello F, Tardella L (2014). Tomato genome-wide transcriptional responses to *Fusarium* wilt and *Tomato Mosaic Virus*. PLoS ONE.

[CR4] Andolfo G, Jupe F, Witek K (2014). Defining the full tomato NB-LRR resistance gene repertoire using genomic and cDNA RenSeq. BMC Plant Biol.

[CR5] Andolfo G, Di Donato A, Darrudi R (2017). Draft of Zucchini (*Cucurbita pepo* L.) proteome: a resource for genetic and genomic studies. Front Genet.

[CR6] Andolfo G, Di Donato A, Chiaiese P (2019). Alien domains shaped the modular structure of plant NLR proteins. Genome Biol Evol.

[CR7] Andolfo G, Villano C, Errico A (2020). Inferring RPW8-NLRs’s evolution patterns in seed plants: case study in *Vitis vinifera*. Planta.

[CR8] Andolfo G, D’agostino N, Frusciante L, Ercolano MR (2021). The tomato interspecific NB-LRR gene arsenal and its impact on breeding strategies. Genes (basel).

[CR9] Bailey TL, Williams N, Misleh C, Li WW (2006). MEME: Discovering and analyzing DNA and protein sequence motifs. Nucl Acids Res.

[CR10] Barchi L, Pietrella M, Venturini L (2019). A chromosome-anchored eggplant genome sequence reveals key events in *Solanaceae* evolution. Sci Rep.

[CR11] Barrera-Redondo J, Ibarra-Laclette E, Vázquez-Lobo A (2019). The genome of *Cucurbita argyrosperma* (silver-seed gourd) reveals faster rates of protein-coding gene and long noncoding RNA turnover and neofunctionalization within *Cucurbita*. Mol Plant.

[CR12] Barrera-Redondo J, Sánchez-de la Vega G, Aguirre-Liguori JA (2021). The domestication of *Cucurbita argyrosperma* as revealed by the genome of its wild relative. Hortic Res.

[CR13] Bayer PE, Edwards D, Batley J (2018). Bias in resistance gene prediction due to repeat masking. Nat Plants.

[CR14] Blanca J, Cañizares J, Roig C (2011). Transcriptome characterization and high throughput SSRs and SNPs discovery in *Cucurbita pepo* (Cucurbitaceae). BMC Genom.

[CR15] Bonardi V, Tang S, Stallmann A (2017). Correction: Expanded functions for a family of plant intracellular immune receptors beyond specific recognition of pathogen effectors. Proc Natl Acad Sci USA.

[CR16] Capuozzo C, Formisano G, Iovieno P (2017). Inheritance analysis and identification of SNP markers associated with *ZYMV* resistance in *Cucurbita pepo*. Mol Breed.

[CR17] Castellanos-Morales G, Paredes-Torres LM, Gámez N (2018). Historical biogeography and phylogeny of *Cucurbita*: insights from ancestral area reconstruction and niche evolution. Mol Phylogenet Evol.

[CR18] D’Esposito D, Cappetta E, Andolfo G (2019). Deciphering the biological processes underlying tomato biomass production and composition. Plant Physiol Biochem.

[CR71] De Bie T, Cristianini N, Demuth JP, Hahn MW (2006). CAFE: a computational tool for the study of gene family evolution. Bioinformatics.

[CR19] Delport W, Poon AFY, Frost SDW, Kosakovsky Pond SL (2010). Datamonkey 2010: a suite of phylogenetic analysis tools for evolutionary biology. Bioinformatics.

[CR20] Di Donato A, Andolfo G, Ferrarini A (2017). Investigation of orthologous pathogen recognition gene-rich regions in solanaceous species. Genome.

[CR21] Dogimont C, Chovelon V, Pauquet J (2014). The *Vat* locus encodes for a CC-NBS-LRR protein that confers resistance to *Aphis gossypii* infestation and *A. gossypii*-mediated virus resistance. Plant J.

[CR22] Finn RD, Clements J, Eddy SR (2011). HMMER web server: interactive sequence similarity searching. Nucleic Acids Res.

[CR23] Garcia-Mas J, Benjak A, Sanseverino W (2012). The genome of melon (*Cucumis melo* L.). Proc Natl Acad Sci USA.

[CR24] Guo YL, Fitz J, Schneeberger K (2011). Genome-wide comparison of nucleotide-binding site-leucine-rich repeat-encoding genes in *Arabidopsis*. Plant Physiol.

[CR25] Guo J, Xu W, Hu Y (2020). Phylotranscriptomics in Cucurbitaceae reveal multiple whole-genome duplications and key morphological and molecular innovations. Mol Plant.

[CR72] Han MV, Thomas GWC, Lugo-Martinez J, Hahn MW (2013). Estimating gene gain and loss rates in the presence of error in genome assembly and annotation using CAFE 3. Mol Biol Evol.

[CR26] Harris KR, Wechter WP, Levi A (2009). Isolation, sequence analysis, and linkage mapping of nucleotide binding site-leucine-rich repeat disease resistance gene analogs in watermelon. J Am Soc Hortic Sci.

[CR27] Jia YX, Yuan Y, Zhang Y (2015). Extreme expansion of NBS-encoding genes in *Rosaceae*. BMC Genet.

[CR28] Jones DT, Taylor WR, Thornton JM (1992). The rapid generation of mutation data matrices from protein sequences. Bioinformatics.

[CR29] Jones P, Binns D, Chang HY (2014). InterProScan 5: genome-scale protein function classification. Bioinformatics.

[CR30] Joobeur T, King JJ, Nolin SJ (2004). The fusarium wilt resistance locus *Fom-2* of melon contains a single resistance gene with complex features. Plant J.

[CR31] Joshi RK, Nayak S (2013). Perspectives of genomic diversification and molecular recombination towards *R*-gene evolution in plants. Physiol Mol Biol Plants.

[CR32] Kang YJ, Kim KH, Shim S (2012). Genome-wide mapping of NBS-LRR genes and their association with disease resistance in soybean. BMC Plant Biol.

[CR33] Kates HR, Soltis PS, Soltis DE (2017). Evolutionary and domestication history of *Cucurbita* (pumpkin and squash) species inferred from 44 nuclear loci. Mol Phylogenet Evol.

[CR34] Katoh K, Misawa K, Kuma K, Miyata T (2002). MAFFT: a novel method for rapid multiple sequence alignment based on fast Fourier transform. Nucl Acids Res.

[CR35] Khoury CK, Carver D, Kates HR (2020). Distributions, conservation status, and abiotic stress tolerance potential of wild cucurbits ( *Cucurbita* L.). Plants People Planet.

[CR36] Kumar S, Stecher G, Tamura K (2016). MEGA7: molecular evolutionary genetics analysis version 7.0 for bigger datasets. Mol Biol Evol.

[CR37] Larkin MA, Blackshields G, Brown NP (2007). Clustal W and Clustal X version 2.0. Bioinformatics.

[CR38] Lin X, Zhang Y, Kuang H, Chen J (2013). Frequent loss of lineages and deficient duplications accounted for low copy number of disease resistance genes in Cucurbitaceae. BMC Genom.

[CR39] Madeira F, Park YM, Lee J (2019). The EMBL-EBI search and sequence analysis tools APIs in 2019. Nucl Acids Res.

[CR40] Marchler-Bauer A, Bryant SH (2004). CD-Search: protein domain annotations on the fly. Nucl Acids Res.

[CR41] McHale L, Tan X, Koehl P, Michelmore RW (2006). Plant NBS-LRR proteins: adaptable guards. Genome Biol.

[CR42] Meyers BC, Kozik A, Griego A (2003). Genome-wide analysis of NBS-LRR–encoding genes in *Arabidopsis*. Plant Cell.

[CR43] Michelmore RW, Christopoulou M, Caldwell KS (2013). Impacts of resistance gene genetics, function, and evolution on a durable future. Annu Rev Phytopathol.

[CR44] Montero-Pau J, Blanca J, Bombarely A (2018). De novo assembly of the zucchini genome reveals a whole-genome duplication associated with the origin of the *Cucurbita* genus. Plant Biotechnol J.

[CR45] Moreira X, Abdala-Roberts L, Gols R, Francisco M (2018). Plant domestication decreases both constitutive and induced chemical defences by direct selection against defensive traits. Sci Rep.

[CR46] Nguyen QM, Iswanto ABB, Son GH, Kim SH (2021). Recent advances in effector-triggered immunity in plants: new pieces in the puzzle create a different paradigm. Int J Mol Sci.

[CR47] Obrero Á, Die JV, Román B (2011). Selection of reference genes for gene expression studies in zucchini (*Cucurbita pepo*) using qPCR. J Agric Food Chem.

[CR48] Ortiz D, de Guillen K, Cesari S (2017). Recognition of the *Magnaporthe oryzae* effector AVR-Pia by the decoy domain of the rice NLR immune receptor *RGA5*. Plant Cell.

[CR49] Osuna-Cruz CM, Paytuvi-Gallart A, Di Donato A (2018). PRGdb 3.0: a comprehensive platform for prediction and analysis of plant disease resistance genes. Nucl Acids Res.

[CR50] Peart JR, Mestre P, Lu R (2005). *NRG1*, a CC-NB-LRR protein, together with N, a TIR-NB-LRR protein, mediates resistance against tobacco mosaic virus. Curr Biol.

[CR73] Pond KSL, Frost SDW (2005). Datamonkey: rapid detection of selective pressure on individual sites of codon alignments. Bioinformatics.

[CR51] Renner SS, Schaefer H (2016). Phylogeny and Evolution of the Cucurbitaceae. Genet Genom Cucurbitaceae.

[CR52] Richly E, Kurth J, Leister D (2002). Mode of amplification and reorganization of resistance genes during recent *Arabidopsis thaliana* evolution. Mol Biol Evol.

[CR53] Román B, Gómez P, Picó B, Die JV (2020). The NBS-LRR gene class is a small family in *Cucurbita pepo*. Preprints.

[CR54] Sanjur OI, Piperno DR, Andres TC, Wessel-Beaver L (2002). Phylogenetic relationships among domesticated and wild species of *Cucurbita* (Cucurbitaceae) inferred from a mitochondrial gene: Implications for crop plant evolution and areas of origin. Proc Natl Acad Sci USA.

[CR55] Sarris PF, Cevik V, Dagdas G (2016). Comparative analysis of plant immune receptor architectures uncovers host proteins likely targeted by pathogens. BMC Biol.

[CR56] Schmittgen TD, Livak KJ (2008). Analyzing real-time PCR data by the comparative CT method. Nat Protoc.

[CR57] Shao ZQ, Xue JY, Wu P (2016). Large-scale analyses of angiosperm nucleotide-binding site-leucine-rich repeat genes reveal three anciently diverged classes with distinct evolutionary patterns. Plant Physiol.

[CR58] Smith BD (1997). The initial domestication of *Cucurbita pepo* in the Americas 10,000 years ago. Science (80-).

[CR59] Soltis PS, Soltis DE (2016). Ancient WGD events as drivers of key innovations in angiosperms. Curr Opin Plant Biol.

[CR60] Sun H, Wu S, Zhang G (2017). Karyotype stability and unbiased fractionation in the paleo-allotetraploid *Cucurbita* genomes. Mol Plant.

[CR61] Tamura K, Nei M, Kumar S (2004). Prospects for inferring very large phylogenies by using the neighbor-joining method. Proc Natl Acad Sci USA.

[CR62] Tian D, Traw MB, Chen JQ (2003). Fitness costs of R-gene-mediated resistance in *Arabidopsis thaliana*. Nature.

[CR63] Untergasser A, Nijveen H, Rao X (2007). Primer3Plus, an enhanced web interface to Primer3. Nucl Acids Res.

[CR64] Wan H, Yuan W, Ye Q (2012). Analysis of TIR- and non-TIR-NBS-LRR disease resistance gene analogous in pepper: characterization, genetic variation, functional divergence and expression patterns. BMC Genom.

[CR65] Wang Z, Gerstein M, Snyder M (2009). RNA-Seq: a revolutionary tool for transcriptomics. Nat Rev Genet.

[CR66] Wu C-H, Derevnina L, Kamoun S (2018). Receptor networks underpin plant immunity. Science (80-).

[CR67] Xanthopoulou A, Montero-Pau J, Mellidou I (2019). Whole-genome resequencing of *Cucurbita pepo* morphotypes to discover genomic variants associated with morphology and horticulturally valuable traits. Hortic Res.

[CR68] Yang S, Feng Z, Zhang X (2006). Genome-wide investigation on the genetic variations of rice disease resistance genes. Plant Mol Biol.

[CR69] Zhang N, Zeng L, Shan H, Ma H (2012). Highly conserved low-copy nuclear genes as effective markers for phylogenetic analyses in angiosperms. New Phytol.

[CR70] Zheng Y, Wu S, Bai Y (2019). Cucurbit Genomics Database (CuGenDB): a central portal for comparative and functional genomics of cucurbit crops. Nucl Acids Res.

