# Supplementary figures and images for: Large-scale gene gains and losses molded the NLR defense arsenal during the Cucurbita evolution
Source: Planta. 2021 Sep 24;254(4):82. doi: 10.1007/s00425-021-03717-x (PMC8463517; doi:10.1007/s00425-021-03717-x)

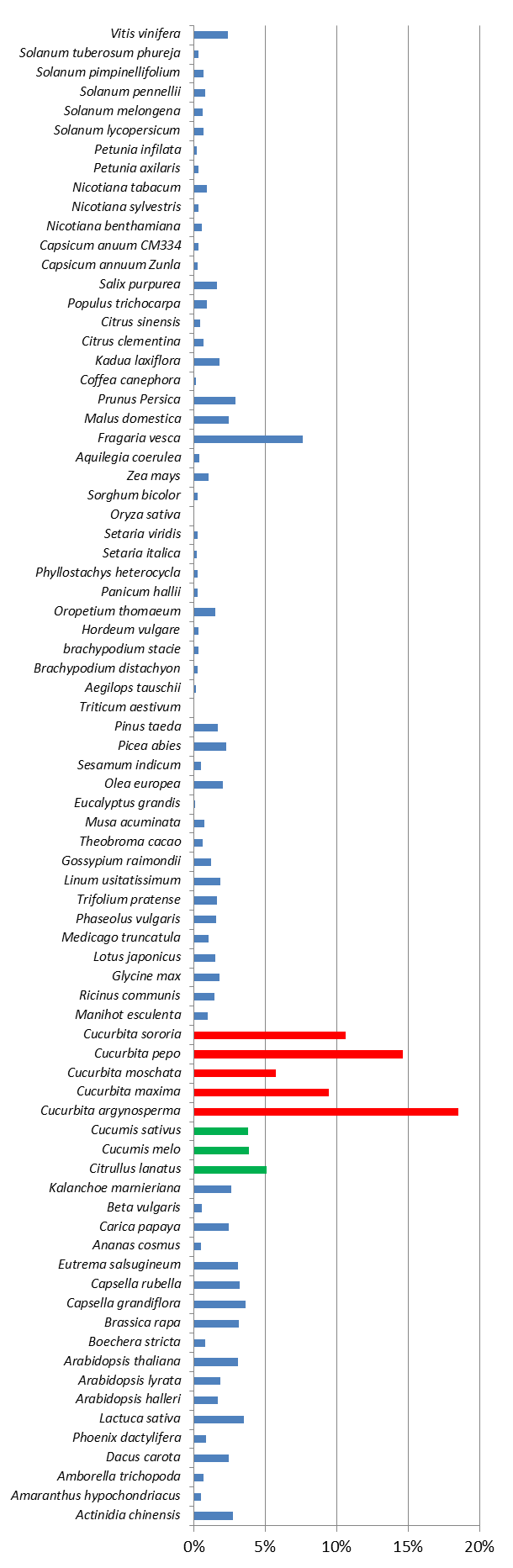

Supplement: Supplementary file 1 — Supplementary file1 Suppl. Fig. S1 Overview of RNL gene subfamily identified in 78 seed plant genomes. Correlation plot between the number of RNL genes and relative NLRs family size (Cucurbita genomes in red and melon, watermelon and cucumber in green). (TIF 113 KB) [file 425_2021_3717_MOESM1_ESM.tif]

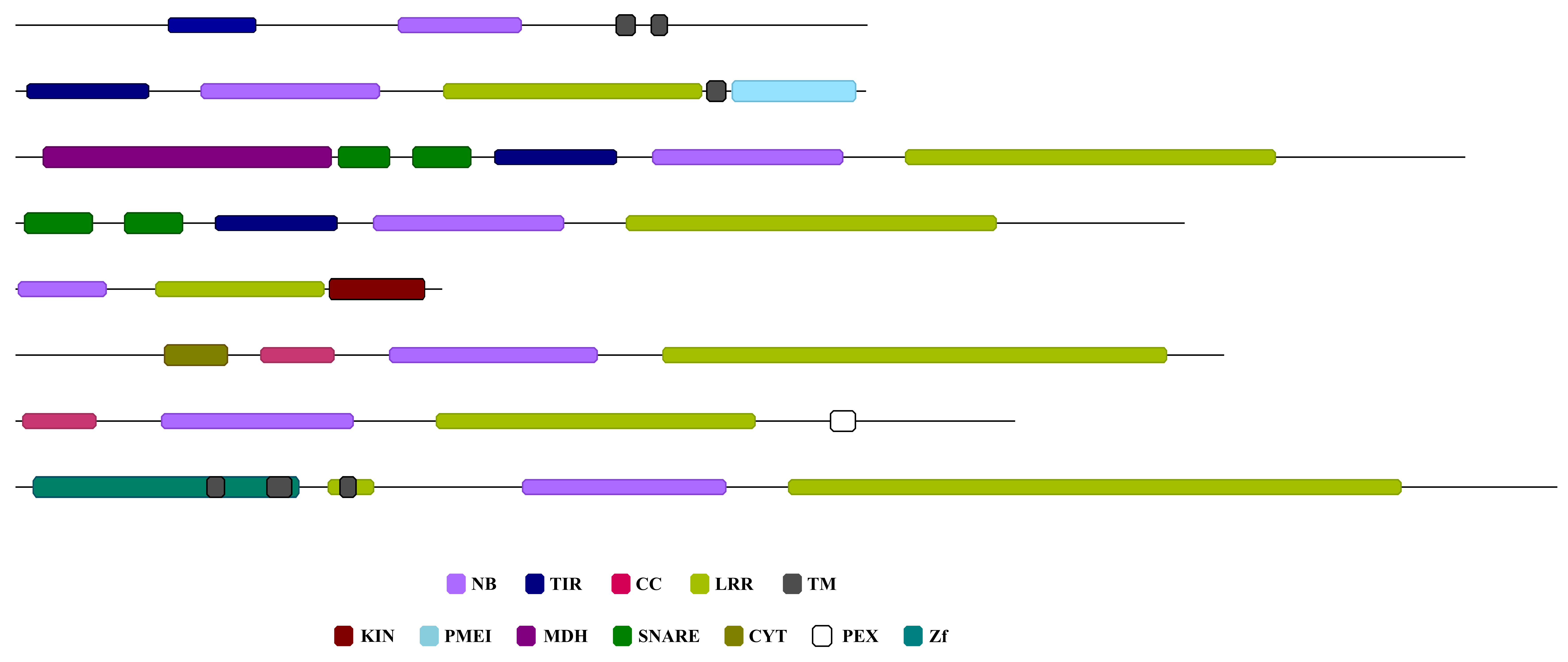

Supplement: Supplementary file 2 — Supplementary file2 Suppl. Fig. S2 Schematic representation of Cucurbita NLR proteins and relative introgressed domains (IDs). MDH: Metallo-dependent hydrolase; SNARE fusion complex; Cytochrome b5-like heme/steroid binding domain; PMEI: pectin methyl-esterase inhibitor; PEX: Pex2-Pex12 domain; Kin: kinase domain; Zf: zinc finger-like domain; TM: transmebrane domain. (TIFF 208 KB) [file 425_2021_3717_MOESM2_ESM.tiff]

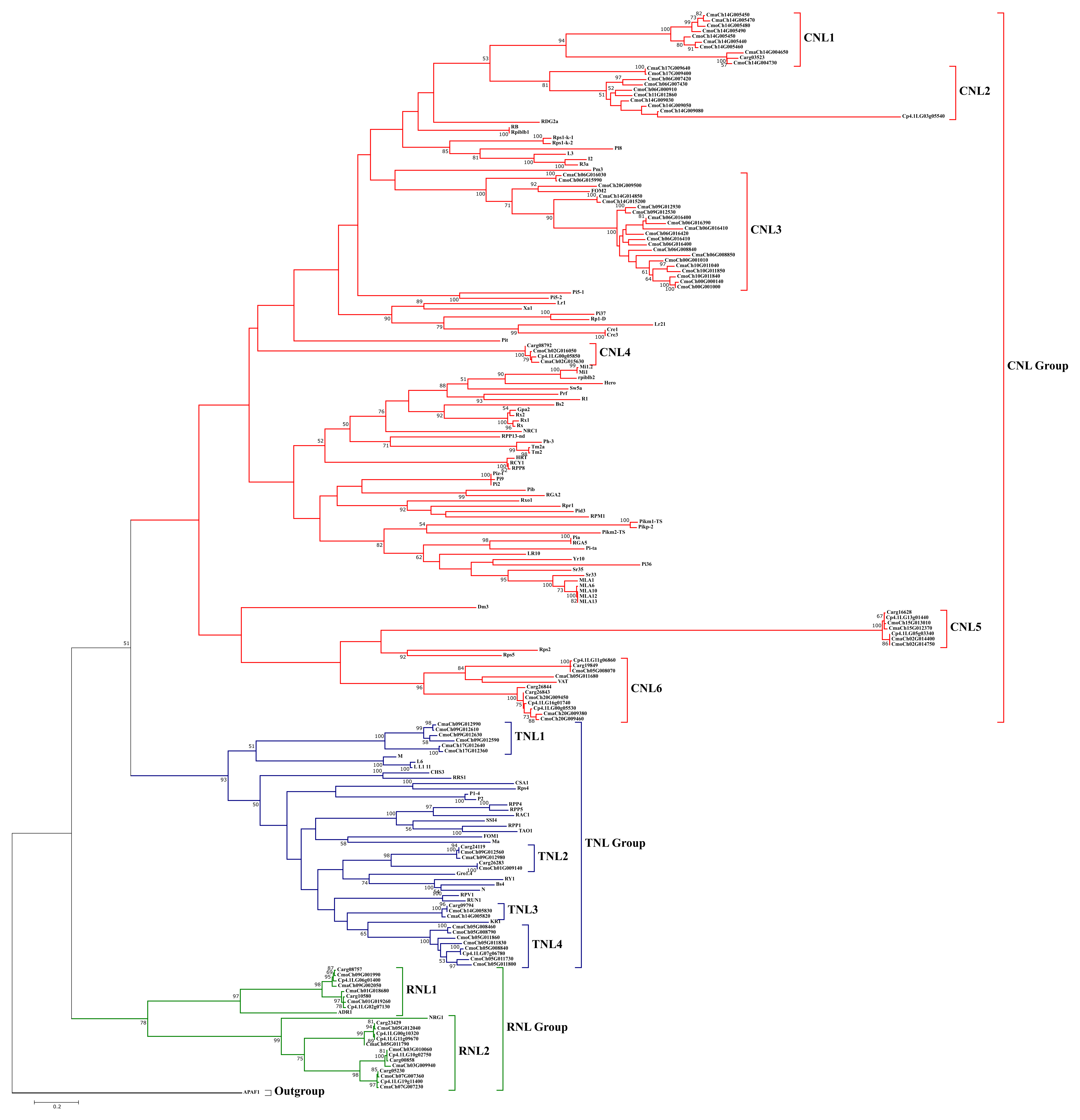

Supplement: Supplementary file 3 — Supplementary file3 Suppl. Fig. S3 Phylogenetic analysis of NLR genes identified in C. argyrosperma, C. maxima, C. moschata and C. pepo genome assemblies. The color of phylogenetic groups refers to Fig. 3. (TIFF 1734 KB) [file 425_2021_3717_MOESM3_ESM.tiff]
